# Radiation Recall Pneumonitis with *Pneumocystis jirovecii* Superinfection and Treatment Induced Hyponatremia in a Patient with Non-Small-Cell Lung Cancer

**DOI:** 10.3390/diseases13110357

**Published:** 2025-11-04

**Authors:** Aleksandra Piórek, Adam Płużański, Dariusz M. Kowalski, Maciej Krzakowski

**Affiliations:** Department of Lung Cancer and Thoracic Tumors, Maria Skłodowska-Curie National Research Institute of Oncology, 02-781 Warsaw, Poland; adam.pluzanski@nio.gov.pl (A.P.); dariusz.kowalski@nio.gov.pl (D.M.K.); maciej.krzakowski@nio.gov.pl (M.K.)

**Keywords:** radiation recall pneumonitis, pembrolizumab, pneumocystis pneumonia, trimethoprim/sulfamethoxazole, hyponatremia, case report

## Abstract

Immune checkpoint inhibitors (ICIs) and thoracic radiotherapy are standard treatments for advanced non-small-cell lung cancer (NSCLC), especially in patients with high PD-L1 expression or symptoms such as superior vena cava syndrome (SVCS). Both therapies carry a risk of pulmonary toxicity, which may be exacerbated by opportunistic infections due to corticosteroid use. We report a unique case of a 65-year-old man with squamous-cell NSCLC and high PD-L1 expression (80%), who developed a rare complication: radiation recall pneumonitis (RRP), with superimposed *Pneumocystis jirovecii* pneumonia and severe symptomatic hyponatremia induced by trimethoprim/sulfamethoxazole (TMP-SMX). The coexistence of these three complications—radiotherapy- and immunotherapy-associated lung injury, opportunistic infection, and electrolyte imbalance—represents an exceptional clinical scenario not previously described in the literature. This report highlights the importance of differential diagnosis, early recognition of complications, and close monitoring of electrolytes in NSCLC patients undergoing complex treatment regimens.

## 1. Introduction

Immunotherapy with immune checkpoint inhibitors (ICIs) has become the backbone of treatment for non-small-cell lung cancer (NSCLC), particularly in patients with high programmed death ligand 1 (PD-L1) expression. Thoracic radiotherapy is a standard modality in symptom-driven cases, such as superior vena cava syndrome (SVCS). However, both therapeutic approaches are associated with pulmonary toxicity, including immune-related pneumonitis and radiation recall pneumonitis (RRP)—a rare phenomenon of inflammatory reactivation within previously irradiated lung tissue, triggered by systemic agents such as ICIs [1,2].

Corticosteroids, used in the management of pneumonitis, increase susceptibility to opportunistic infections. One of the most serious is *Pneumocystis jirovecii* pneumonia (PJP), historically associated with HIV infection but increasingly recognized in non-HIV immunocompromised patients, including those receiving corticosteroids for cancer-related toxicities [3,4].

Trimethoprim-sulfamethoxazole (TMP-SMX), the drug of choice in the treatment of PJP, has been associated with serious electrolyte disturbances, including severe hyponatremia and hyperkalemia. These adverse effects are attributed to trimethoprim’s amiloride-like activity, which blocks epithelial sodium channels in the distal nephron, mimicking a syndrome of inappropriate antidiuretic hormone secretion (SIADH) [5,6,7].

To our knowledge, this is the first reported case of a patient with NSCLC who developed radiation recall pneumonitis following ICI therapy, superinfected with PJP, and who experienced severe symptomatic hyponatremia induced by TMP-SMX. The co-occurrence of these three complications, each serious and rarely overlapping, underscores the complexity of care in patients undergoing multimodal cancer therapy and highlights the need for vigilance in differential diagnosis and electrolyte monitoring.

## 2. Case Presentation

A 65-year-old male, former heavy smoker (20 cigarettes daily for 30 years), with no documented history of chronic pulmonary disease, presented with progressive dyspnea, hemoptysis, cough, and hoarseness. He had a known medical history of arterial hypertension, atherosclerosis, venous thrombosis, and past percutaneous transluminal angioplasty of the right lower limb. There was no reported family history of lung cancer. The patient declined to provide details regarding his environmental and psychosocial background. His performance status on the ECOG scale was 1.

A computed tomography (CT) scan of the chest revealed a large right hilar mass with mediastinal lymphadenopathy. Bronchoscopy with biopsy confirmed squamous-cell NSCLC with high programmed death ligand 1 (PD-L1) expression (80%). Oncologic staging classified the tumor as T4N2Mx, according to the 8th edition of the TNM classification by the Union for International Cancer Control (UICC) and the International Association for the Study of Lung Cancer (IASLC). Prognosis was assessed as poor due to advanced locoregional disease. The patient was qualified for palliative treatment.

**Figure 1:**
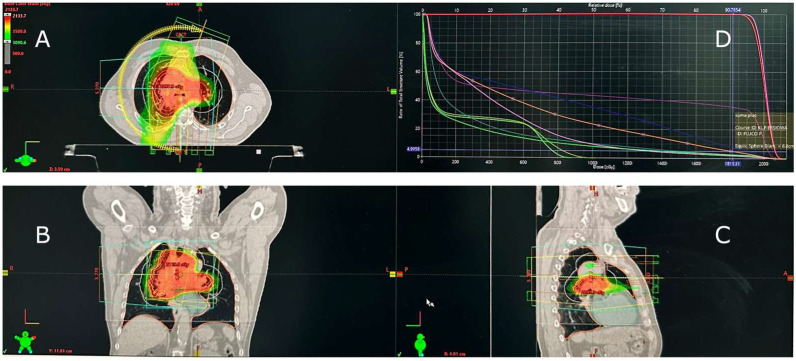
Radiotherapy treatment plan for superior vena cava syndrome (SVCS) caused by squamous-cell lung cancer. (**A**) Axial, (**B**) coronal, and (**C**) sagittal CT views demonstrate conformal dose distribution within the mediastinal target. (**D**) Dose–volume histogram (DVH) illustrates low pulmonary exposure. Treatment was delivered using volumetric modulated arc therapy (VMAT). The red, orange, yellow, and green areas represent decreasing isodose levels, and the curves in panel D illustrate dose–volume relationships for the target and organs at risk.

Due to symptomatic SVCS, the patient underwent palliative radiotherapy (30 Gy in 10 fractions) delivered with volumetric modulated arc therapy (VMAT) to achieve rapid symptom relief and a conformal dose distribution with minimal treatment time. The mean lung dose (MLD) was 5.82 Gy, the lung volume receiving 20 Gy (V20) was 1%, and the maximum lung dose (Dmax) was 18.15 Gy (Figure 1).

Given the high PD-L1 expression, pembrolizumab monotherapy (200 mg i.v. every 3 weeks) was initiated. Initial cycles led to clinical improvement, but after the third infusion, the patient developed worsening dyspnea, dry cough, and fever. CT imaging revealed ground-glass opacities and interstitial consolidations, primarily in the irradiated lung field. Radiation and immune-related pneumonitis were diagnosed. High-dose corticosteroid therapy was initiated with intravenous methylprednisolone at 2 mg/kg/day (approximately 140 mg/day). The dose was reduced to 1.5 mg/kg/day after five days and subsequently tapered to 1 mg/kg/day over the following two weeks, with gradual transition to oral prednisone, with clinical improvement.

Four weeks after initiation of corticosteroid therapy, the patient developed new symptoms in form of low-grade fever and dry cough. Bronchoalveolar lavage (BAL) fluid analysis revealed a total cell count of 180/μL, with 45% macrophages, 42% lymphocytes, 10% neutrophils, and 3% eosinophils. The CD4/CD8 ratio was 1.1. PCR testing detected *Pneumocystis jirovecii* DNA, while bacterial and viral panels (including Mycoplasma pneumoniae, Chlamydophila pneumoniae, CMV, HSV, and adenovirus) were negative. This cytological and molecular profile was consistent with *Pneumocystis jirovecii* pneumonia in an immunocompromised non-HIV patient, without additional evidence of immune-mediated pneumonitis activity. On admission, the patient presented with a respiratory rate of 22 breaths/min, oxygen saturation of 87% on ambient air, and body temperature of 36.4 °C. Laboratory tests revealed elevated inflammatory markers: CRP 213 mg/L and LDH 521 IU/L. The leukocyte count was 8.78 × 10^9^/L. β-D-glucan was significantly elevated at 218 pg/mL, supporting the diagnosis of *Pneumocystis jirovecii* pneumonia (PJP). Therapeutic-dose trimethoprim/sulfamethoxazole (TMP-SMX) was initiated. On day 10 of therapy, the patient developed symptomatic hyponatremia (serum sodium 122 mmol/L) with progressive deterioration. TMP-SMX was administered at a total daily dose corresponding to approximately 15 mg/kg of TMP and 75 mg/kg of SMX, in line with current treatment recommendations for *Pneumocystis jirovecii* pneumonia. For the first five days, a combination of intravenous and oral therapy was used (Trimesolphar 480 mg/5 mL, 4–6 ampoules daily, and Biseptol 960 mg, three times daily) to maintain effective drug concentrations while avoiding fluid overload. Subsequently, due to fluid restriction requirements, the regimen was switched to full oral treatment (Biseptol 960 mg, two tablets three times daily).

Serum sodium levels progressively declined from 130 mmol/L at baseline to a nadir of 106 mmol/L, accompanied by disorientation, somnolence, and dizziness. At the time of serum sodium nadir, urine sodium concentration was markedly elevated (181 mmol/L) and urine osmolality was 603 mOsm/kg, while serum osmolality was 245 mOsm/kg. This pattern was consistent with inappropriate renal sodium loss and impaired free water excretion, supporting a TMP-SMX–induced mechanism rather than dilutional hyponatremia. Following discontinuation of TMP-SMX, intravenous 3% sodium chloride and fluid restriction were implemented. Serum sodium gradually increased to 121 mmol/L after four days and normalized to 131 mmol/L within ten days, with complete clinical recovery.

Following clinical stabilization, the patient was enrolled in home palliative care. Follow-up imaging showed no evidence of tumor progression. Follow-up PCR testing for *Pneumocystis jirovecii* was performed one month after the completion of antibiotic therapy and the patient’s discharge from the hospital; the result was negative. The patient was monitored with CT scans performed every three months. Three months after hospital discharge, radiographic progression was observed. Molecular analysis confirmed the presence of a KRAS G12C mutation, and second-line treatment with sotorasib was initiated. The patient received sotorasib for three months; however, subsequent CT imaging revealed further disease progression. In light of both radiological and clinical progression, with deterioration to an ECOG performance status of 3, the patient was referred to best supportive care (BSC). No further systemic anticancer therapy was administered.

**Figure 2:**
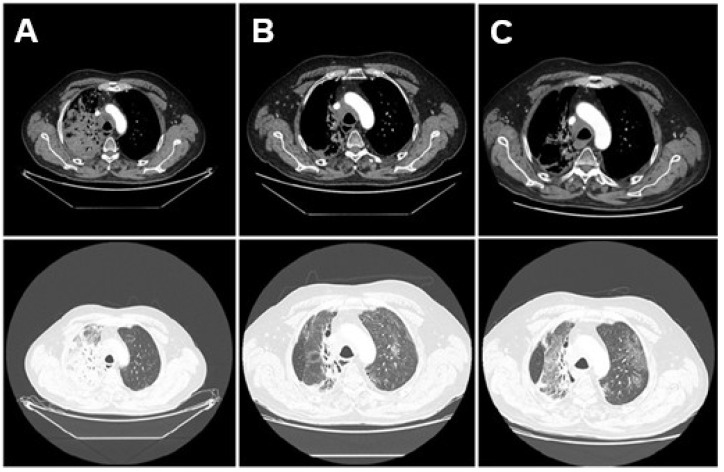
Sequential axial chest CT scans illustrating the evolution of lung findings. Mediastinal (**top**) and lung window (**bottom**). (**A**) CT from 10 March 2025, prior to treatment, showing a right hilar tumor and no inflammatory lung changes. (**B**) CT from 7 May 2025, during hospitalization, revealing bilateral ground-glass opacities and consolidations consistent with pneumonitis and Pneumocystis jirovecii pneumonia. (**C**) CT from 20 May 2025, showing improvement in pulmonary opacities after treatment with corticosteroids and trimethoprim-sulfamethoxazole.

The evolution of radiological findings—initial pneumonitis limited to the irradiated field, subsequent development of bilateral ground-glass opacities consistent with PJP, and resolution after antimicrobial treatment—is illustrated in Figure 2.

## 3. Discussion

### 3.1. Radiation Recall Pneumonitis Syndrome and Immunotherapy

#### 3.1.1. Incidence of Radiation Recall Pneumonitis Following Immunotherapy

Radiation recall pneumonitis (RRP) is a rare complication of oncologic treatment. While historically associated with chemotherapy or targeted agents, recent years have seen increasing reports of RRP triggered by immune checkpoint inhibitors (ICIs) [8,9,10]. Its true incidence remains uncertain. Cousin et al. (2021) reported RRP in 18.8% of lung cancer patients treated with PD-1/PD-L1 inhibitors after prior radiotherapy [9]. The median time from radiotherapy to RRP onset was approximately 15 months (range: 7.5 months to 5 years). Other analyses also indicate RRP is relatively rare, with one Chinese cohort reporting a 7% incidence [11]. Despite this, most patients treated with ICIs post-radiotherapy do not develop RRP, supporting the overall safety of this sequence under proper monitoring [11].

#### 3.1.2. Mechanism of Radiation Recall Pneumonitis Development

The mechanism of RRP involves an exaggerated inflammatory response in previously irradiated lung tissue, triggered by systemic therapy [10,12]. Radiotherapy causes initial cell damage and local inflammation that typically resolves in weeks or months. However, a pro-inflammatory microenvironment may persist in the irradiated area (e.g., fibrosis with chronic presence of pro-inflammatory cytokines) [10,11,12,13]. The use of immunotherapy—which stimulates T lymphocytes to damage cancer cells—may simultaneously “unlock” an immune response directed against antigens or cells within the radiation-damaged region of the lung [12]. As a result, an acute inflammatory relapse occurs within the irradiated field, resembling classic acute radiation pneumonitis (hence the term “recall”) [10].

Notably, several patients who develop RRP have shown concurrent clinical benefit from immunotherapy—reported cases often featured a good tumor response to treatment [8,13,14,15,16]. This suggests that a strong activation of the immune system, which combats the tumor, may paradoxically also promote the reactivation of radiation-induced lung damage. A similar course was observed in our patient—he achieved a partial response (PR) to immunotherapy and showed no disease progression despite treatment discontinuation; unfortunately, our patient developed RRP.

It also appears that the type of immunotherapy used influences the risk of RRP. It has been reported that anti–PD-1 antibodies more frequently cause immune-related pneumonitis than anti–PD-L1 antibodies [9,11,17]. Possibly, PD-1 blockade more strongly enhances T-cell infiltration (as it blocks interactions with both PD-L1 and PD-L2), which translates into a higher tendency for such reactions [11]. In one study, no significant correlations were found between RT dosimetric parameters and the occurrence of RRP [9], although others suggest that larger irradiated lung volumes (higher V5, V20, and MLD) may increase the risk [8,11,12]. In the analysis by Lu et al. (2022), additional risk factors for RRP were identified: age < 65 years (HR 6.24), presence of chronic lung disease (HR 6.64), and fibrotic changes on CT prior to starting immunotherapy (HR 9.13), which may help identify patients particularly vulnerable to this complication [11].

#### 3.1.3. Diagnosis of RRP

Diagnosing RRP is challenging and is based on clinical and imaging correlation. The classic triad includes (1) inflammatory changes confined to the prior radiation field, (2) delayed onset beyond the acute RT reaction window (typically ≥6 months post-radiation), and (3) temporal association with systemic drug exposure [8,10,12,16]. The interval between radiotherapy and the onset of RRP is highly variable—it may range from a few weeks to several months (especially if immunotherapy is initiated soon after irradiation), but recall can also occur many years later (cases have been reported 4–5 years after completion of radiotherapy) [10,11,12,18]. According to the classic Camidge and Price criteria, a reaction is considered RRP only if it occurs ≥7 days after radiotherapy, which helps distinguish it from acute radiation-induced toxicity [12].

The clinical presentation of RRP is difficult to distinguish from other forms of pneumonitis: patients may exhibit dyspnea, cough, sometimes low-grade fever or chest pain, or—in rarer cases—remain asymptomatic with findings detectable only on CT imaging [8,10,12]. A thorough clinical history is crucial: prior thoracic radiotherapy combined with recent initiation (or rechallenge) of immunotherapy supports the diagnosis [10,12].

Imaging studies play a crucial role in diagnosis. Chest X-rays may reveal opacities in the previously irradiated area [12], but the gold standard is high-resolution CT (HRCT). Typically, HRCT shows focal inflammatory changes confined to the previously irradiated area, often presenting as ground-glass opacities, interstitial reticular changes, or consolidations with air bronchograms [2,10,11,12].

In the differential diagnosis, it is essential to distinguish RRP primarily from immune-related pneumonitis which is not confined to the irradiated field. It is a common complication of immunotherapy but usually involves structures beyond the radiation area—unlike the focal nature of RRP [2,8,10].

Additionally, pulmonary infection must be considered, especially in immunosuppressed patients. Microbiological tests (cultures, tests for atypical pathogens) are performed along with the assessment of clinical signs such as fever and leukocytosis [2,8,10]. Infectious pneumonia may mimic RRP radiologically but typically does not respect the boundaries of the previous radiation field and may follow a different clinical course (e.g., responds to antibiotics).

Another important consideration is differentiating inflammatory infiltrates from potential tumor progression. Within the irradiated field, infiltrates can be mistaken for tumor regrowth—particularly in lung cancer, where inflammatory changes may resemble infiltrative tumor recurrence [10].

In summary, RRP is a diagnosis of exclusion—it is made after ruling out infection and other pulmonary causes, in a patient presenting with typical infiltrates localized to the former radiation field and a history of receiving a triggering drug [11,12,19].

#### 3.1.4. Treatment of RRP

Therapeutic management of RRP is primarily based on clinical experience from treating radiation-induced and immune-related pneumonitis, as there are currently no specific guidelines for RRP. The initial and essential step is discontinuation of the immunotherapy agent that triggered the reaction [10,12]. The standard treatment involves initiating systemic corticosteroids, similar to protocols used for pneumonitis following radiotherapy or immunotherapy. High-dose steroids are recommended—such as prednisone/prednisolone at 1–2 mg/kg/day, or intravenous methylprednisolone in cases of more severe presentation—with a slow tapering schedule over several weeks [2,8,12]. In general, corticosteroids should be continued for at least 4–6 weeks with gradual dose reduction to prevent recurrence of inflammation [2,12].

In addition to corticosteroids, supportive treatment is also employed: oxygen therapy in cases of significant hypoxemia, bronchodilators if bronchial obstruction is present, and prophylaxis or treatment of infections (antibiotics) until an infectious cause is ruled out [10,12].

An important consideration is the continuation of oncologic therapy after the resolution of RRP. Reintroduction of immunotherapy requires a careful assessment of risks and benefits. It is suggested that if the RRP episode was mild and promptly controlled, and continuation of immunotherapy offers meaningful oncologic benefits, cautious rechallenge may be considered under close clinical monitoring [2,8,11,12].

### 3.2. Pneumocystis jirovecii Infection (PCP)

*Pneumocystis jirovecii* infection is an opportunistic fungal lung infection that, in immunocompromised individuals—particularly those treated with corticosteroids, transplant recipients, or patients with HIV—can lead to severe, sometimes life-threatening pneumonia [20,21]. Although pneumocystosis has been less frequently reported following radiotherapy, it may occur, especially in the context of radiation-related complications such as pneumonitis, and particularly after RRP triggered by immunotherapy and treated with glucocorticosteroids. This condition may predispose patients to secondary *P. jirovecii* infection [22].

The clinical presentation includes fever, exertional dyspnea, dry cough, and a characteristic CT finding of ground-glass opacities [20,21].

#### 3.2.1. Diagnosis of PCP

The diagnosis of PCP is based on the detection of the pathogen in respiratory specimens (BAL, induced sputum) using microscopic methods (immunofluorescence) or molecular techniques (PCR), which now represent the diagnostic standard—particularly PCR from BAL samples [4,20,21].

#### 3.2.2. Treatment of PCP

The treatment of choice remains TMP/SMX, which is effective for both the treatment and prevention of PCP [21]. In more severe cases—or in the presence of hypoxemia (PaO_2_ ≤ 70 mmHg, or an alveolar-arterial gradient ≥ 35 mmHg)—the addition of glucocorticosteroid therapy is recommended [20,21].

#### 3.2.3. Prophylaxis of PCP

Prophylaxis of PCP is essential in patients with moderate to severe immunosuppression, particularly when receiving >20 mg/day of prednisolone for ≥4 weeks [4,22]. Prophylactic dosing of TMP/SMX (e.g., once daily or three times per week) has proven effective in significantly reducing the incidence of PCP [4,20].

### 3.3. Trimethoprim–Sulfamethoxazole-Induced Hyponatremia

Trimethoprim (TMP) acts as a potassium-sparing diuretic by blocking epithelial sodium channels (eNaC) in the distal renal tubules, leading to natriuresis and hyponatremia [6,7]. In patients receiving high doses of TMP-SMX (e.g., for PCP), hyponatremia may affect approximately 72% of hospitalized individuals [23]. Hyponatremia developing during therapy can have various pathophysiological mechanisms—most commonly hypovolemic, due to natriuresis, or related to syndrome of inappropriate antidiuretic hormone secretion (SIADH). Even standard oral doses may cause symptomatic hyponatremia via SIADH [23].

Monitoring serum sodium should be part of routine care in patients treated with TMP-SMX, especially when high doses are used or in those undergoing immunosuppressive therapy [7]. Accurate diagnosis requires the assessment of fluid balance as well as measurements of urine osmolality and electrolyte concentrations. Treatment includes discontinuation of TMP-SMX, correction of sodium deficit, and fluid restriction [23].

## 4. Conclusions

This case illustrates the complexity of pulmonary complications in patients with non-small-cell lung cancer receiving multimodal therapy. Radiation-induced and immune-related pneumonitis may overlap and evolve into life-threatening conditions, particularly when compounded by opportunistic infections such as *Pneumocystis jirovecii* pneumonia. Clinicians should maintain a high index of suspicion for secondary infections in patients receiving high-dose corticosteroids, and consider prophylaxis in at-risk individuals. Furthermore, while trimethoprim-sulfamethoxazole remains the first-line treatment for PCP, its potential for inducing severe electrolyte disturbances, including hyponatremia, must be acknowledged. Regular monitoring of serum sodium and prompt intervention are essential. Interdisciplinary management and early recognition of complications are crucial to ensure optimal patient outcomes.

## Data Availability

All relevant clinical data are included in the article. Additional information is available from the corresponding author upon reasonable request.

## Abbreviations

The following abbreviations are used in this manuscript:

BAL	Bronchoalveolar lavage
CT	Computed tomography
DFS	Disease-free survival
HRCT	High-resolution computed tomography
ICIs	Immune checkpoint inhibitors
MLD	Mean lung dose
NSCLC	Non-small-cell lung cancer
OS	Overall survival
PCP	*Pneumocystis jirovecii* pneumonia
PCR	Polymerase chain reaction
PD-L1	Programmed death-ligand 1
PFS	Progression-free survival
PJP	*Pneumocystis jirovecii* pneumonia
RRP	Radiation recall pneumonitis
SIADH	Syndrome of inappropriate antidiuretic hormone secretion
SVCS	Superior vena cava syndrome
TMP-SMX	Trimethoprim/sulfamethoxazole
VMAT	Volumetric modulated arc therapy
V20	Lung volume receiving ≥20 Gy
